# GENOMIC ARCHITECTURE OF FEMALE REPRODUCTIVE AGING

**DOI:** 10.1093/geroni/igad104.1000

**Published:** 2023-12-21

**Authors:** Anna Murray

**Affiliations:** University of Exeter, Exeter, England, United Kingdom

## Abstract

Female reproductive lifespan is characterised by declining ovarian reserve, but the underlying mechanism is poorly understood. Reproductive ageing is a multifactorial trait with both genetic and environmental modifiers. Our research uses genome-wide analyses to identify the genes and biological processes that govern female reproductive ageing. We have identified 290 genetic loci with relatively common variants (>0.1%) that are associated with timing of natural menopause, in a meta-analysis of >200,000 women in the ReproGen consortium. In combination these common variants explain around 12% of the variation in menopause timing. Women in the top 1% combined genetic risk centile for earlier menopause are approximately 5x more likely to have menopause before 40 years. A high genetic risk for earlier menopause in mothers, but not fathers, is also associated with increased de novo mutation rate in offspring. We assessed the role of rare (MAF<0.1%) protein-coding variants on ovarian function in data from 104,733 females in the UK Biobank using gene burden association tests. We identified nine genes with associations that passed our multiple testing threshold. Protein-truncating variants in ZNF518A were associated with earlier menopause (effect size 5.61 years, P=1.2x10-10), with an effect larger than for any previously reported gene or variant. Deleterious variants in SAMHD1 were associated with >1 year later menopause and were also a germline risk factor for cancer predisposition in males and females. Understanding the genetics of reproductive ageing is not only important for fertility, but also has implications for broader age-related health outcomes, such as cancer, diabetes and osteoporosis.

